# Exploring the host factors affecting asymptomatic *Plasmodium falciparum* infection: insights from a rural Burkina Faso study

**DOI:** 10.1186/s12936-023-04686-0

**Published:** 2023-09-01

**Authors:** Peter J. Neyer, Bérenger Kaboré, Christos T. Nakas, Britta Hartmann, Annelies Post, Salou Diallo, Halidou Tinto, Angelika Hammerer-Lercher, Carlo R. Largiadèr, Andre J. van der Ven, Andreas R. Huber

**Affiliations:** 1 Institute of Laboratory Medicine, Kantonsspital Aarau, Aarau, Switzerland; 2 Graduate School for Cellular & Biomedical Sciences, University of Bern, Bern, Switzerland; 3 Department of Clinical Chemistry, Inselspital, Bern University Hospital, University of Bern, Bern, Switzerland; 4 Department of Internal Medicine, Radboud Center for Infectious Diseases, Radboud University Medical Center, Nijmegen, The Netherlands; 5 IRSS/Clinical Research Unit of Nanoro (CRUN), Nanoro, Burkina Faso; 6 Laboratory of Biometry, Department of Agriculture Crop, Production and Rural Environment, University of Thessaly, Volos, Greece; 7 Private University in the Principality of Liechtenstein, Triesen, Principality of Liechtenstein

**Keywords:** Haemoglobin variants, Malaria, Nutrition

## Abstract

**Background:**

Asymptomatic *Plasmodium falciparum* parasitaemia forms a reservoir for the transmission of malaria disease in West Africa. Certain haemoglobin variants are known to protect against severe malaria infection. However, data on the potential roles of haemoglobin variants and nongenetic factors in asymptomatic malaria infection is scarce and controversial. Therefore, this study investigated the associations of iron homeostasis, inflammation, nutrition, and haemoglobin mutations with parasitaemia in an asymptomatic cohort from a *P. falciparum*-endemic region during the high transmission season.

**Methods:**

A sub-study population of 688 asymptomatic individuals (predominantly children and adolescents under 15 years, n = 516) from rural Burkina Faso previously recruited by the NOVAC trial (NCT03176719) between June and October 2017 was analysed. Parasitaemia was quantified with conventional haemocytometry. The haemoglobin genotype was determined by reverse hybridization assays targeting a selection of 21 *HBA* and 22 *HBB* mutations. Demographics, inflammatory markers (interleukins 6 and 10, hepcidin), nutritional status (mid upper-arm circumference and body mass index), and anaemia (total haemoglobin, ferritin, soluble transferrin receptor) were assessed as potential predictors through logistic regression.

**Results:**

Malaria parasites were detected in 56% of subjects. Parasitaemia was associated most strongly with malnutrition. The effect size increased with malnutrition severity (OR = 6.26, CI_95_: 2.45–19.4, p < 0.001). Furthermore, statistically significant associations (p < 0.05) with age, cytokines, hepcidin and heterozygous haemoglobin S were observed.

**Conclusions:**

According to these findings, asymptomatic parasitaemia is attenuated by haemoglobin S, but not by any of the other detected genotypes. Aside from evidence for slight iron imbalance, overall undernutrition was found to predict parasitaemia; thus, further investigations are required to elucidate causality and inform strategies for interventions.

**Supplementary Information:**

The online version contains supplementary material available at 10.1186/s12936-023-04686-0.

## Background

Malaria is still a large public health concern and a burden on health economies, especially in West Africa. Despite continuing advances in malaria control and elimination [1, 2], such as chemoprevention with small molecules or therapeutic antibodies and recently endorsed vaccines, the World Health Organization (WHO) recorded a striking global death toll for malaria of 619,000 in 2021, of which 95% was counted in Africa [3].

The spread of malaria disease by mosquitoes is dependent on environmental, parasite- and host-related factors such as the amount of precipitation, parasite genotype or host red blood cell variants and immunological control of parasites [4–6]. Transmission is possible in prolonged infection with resulting gametocytaemia [7, 8]. Since asymptomatic infection is a mere incidental finding and normally remains unnoticed and thus untreated, these individuals might carry parasites over months. This asymptomatic malaria parasitaemia forms a reservoir of parasites, which is a major concern since it facilitates transmission of malaria disease [9].

Numerous factors influencing asymptomatic parasite carriage are poorly understood. For instance, certain mutations are overrepresented in malaria-endemic regions compared to where malaria infection is less prevalent [5, 10]. In recent years, several large mapping studies have focused on beta-globin gene (*HBB*) mutations, such as haemoglobin C (AC) or haemoglobin S (AS), and have demonstrated a geographic correlation with these mutations [4, 5, 9]. In particular, AC, AS, and alpha gene (*HBA*) mutations, such as the prevalent 3.7 kb deletion (-a/aa), have been demonstrated to offer some protection against severe *Plasmodium falciparum* malaria infection [10–12]. However, malaria susceptibility is complex and possibly influenced by polygenetic effects [10].

On the other hand, nongenetic factors play an important role, albeit direct or indirect. For example, iron parameters, such as ferritin or hepcidin, are affected by malaria parasite carriage [13] and cytokines [14, 15]. The association of nutrition is controversially discussed, partially due to inappropriate or incomplete anthropometry and lack of standardization [16]. Nevertheless, observational data from West Africa support a crucial relationship between undernutrition and malaria [17].

A large and well-characterised cohort of asymptomatic non-febrile individuals in the *P. falciparum* hyper-endemic rural region Nanoro in Burkina Faso was used to clarify the impact of several possible influencing factors on parasitaemia and parasite density. Among these factors, haemoglobin genotypes as well as several surrogate markers for iron homeostasis (hepcidin, ferritin, soluble transferrin receptor), inflammation (cytokines: interleukin 6, IL-6, and interleukin 10, IL-10), and nutrition were investigated. Furthermore, the abundance of *P. falciparum* parasitaemia is reported in these asymptomatic individuals in this region and its predictors are described.

## Methods

### Study design

For this study, the cohort of the Nonspecific Effects of Vaccination on Mortality and Morbidity (NOVAC) trial (ClinicalTrials.gov Identifier: NCT03176719) was investigated. This large cross-sectional study included healthy volunteers randomly selected from the Nanoro health and demographic surveillance system [18] during the rainy season (from June through October 2017). The survey was conducted among 24 villages with sampling quotas depending on the respective number of inhabitants. The primary goal of this study was to achieve a representative sample to establish haemocytometric reference values. For this reason, 200 subjects for each of five age groups were recruited: between 12 and 23 months, 2 to 4 years, 5 to 9 years, 10 to 14 years, and above 14 years. The detailed design of the original study has been described previously [19].

### Eligibility criteria

Participants were included when neither signs nor symptoms were noticed by the study physician. Eligibility was further tested with the following exclusion criteria: current febrile illness, known chronic illness, HIV, tuberculosis, renal failure, or cardiac disease. Furthermore, individuals who had participated in the concurrent vaccination trial from Glaxo-Smith-Kline (ClinicalTrials.gov Identifier: NCT03143218) at the same inclusion site were excluded.

### Ethics

The study protocols were approved by the national ethics committee of Burkina Faso (ref 2016-01-006). Written informed consent was obtained from all participants or their parents/legal guardians. Assent was obtained from all participants aged 7 to 20 years according to the local requirements.

### Haemoglobin genotyping

Molecular genetic analyses were performed at the Institute of Laboratory Medicine (Aarau, Switzerland) using the α-Globin StripAssay and β-Globin StripAssay MED kits (ViennaLab Diagnostics, Vienna, Austria) to identify 21 and 22 haemoglobin mutations, respectively (detailed list is presented in Additional file 1, 2). Briefly, genomic DNA was extracted from cell pellets that had been frozen (− 20 °C) for approximately 3 years without thawing and refreezing. Samples were thawed at room temperature and vortexed, and a portion (100 µL) was lysed with 400 µL of universal lysis buffer (Seegene). Extraction was automated on a QIAsymphony SP instrument (QIAgen, Hombrechtikon, Switzerland) using proprietary kits and the most common mutations were thus investigated.

### Nongenetic parameters

Demographics were registered, and height, weight, mid upper-arm circumference (MUAC) and body temperature were measured upon inclusion. Malnutrition categorization was performed by calculating z scores for the MUAC-for-age with the “zscorer” package [20] for individuals below 19 years of age and for older individuals by body mass index (BMI). Commonly used cutoffs were applied [21–24] to assign subjects into groups of energy sufficiency (normal) and mild, moderate, or severe malnutrition. The infants, children and adolescents were scored with the MUAC-for-age z score because it was validated in African children and thus most fitting for this cohort [25].

Haemocytometry, including malaria-infected red blood cell and gametocyte quantification, was performed on an XN-30 automated haematology analyser (Sysmex, Kobe, Japan). Anaemia was defined by age-specific cutoffs for total haemoglobin concentrations published by the WHO [26]. Parasitaemia was (A) defined as the occurrence of malaria-infected red blood cells above a given sensitivity detection threshold previously established ([27], > 20 parasites per microlitre from low malaria mode) and (B) quantified in absolute numbers of parasites per microlitre (parasite density). Gametocyte counts were categorised into positive and negative by a cutoff of 1.3 gametocytes per microlitre.

Measurement of inflammatory biomarkers (IL-6, IL-10, tumour necrosis factor, interferon gamma) and iron biomarkers (soluble transferrin receptor, hepcidin, ferritin) was conducted by multiplex immunoassay (MAGPIX, Luminex) and quantitative sandwich immunoassay (Quantakine and Thermo Scientific), respectively.

### Statistical analysis

The Wilcoxon–Mann–Whitney U test was employed for comparisons of location parameters when parametric assumptions were not met. The t test was used for comparison of means after log transforming the data, and the subsequent test for departure from normality was not significant. Univariate and multivariable analyses for the assessment of possible predictors of a binary response (presence of parasitaemia) were carried out through logistic regression using odds ratios to quantify the relationship. 95% confidence intervals for proportions were calculated per category based on binomial distribution theory. Multiple logistic regression was performed on a subset of the dataset where measurements for all the covariates were present. The chi-squared test for trend was used for the assessment of the existence of a linear trend for ordered categorical data vs. a binary outcome. Statistical analyses were performed using R version 4.3.0 (The R Foundation for Statistical Computing, Vienna, Austria). The testwise alpha level was set at 0.05. P values less than 0.05 were considered statistically significant.

## Results

In total, 1012 asymptomatic individuals were included in the original NOVAC study. For the current study, genotyping was performed in 847 individuals with sufficient sample volume for genomic DNA extraction. Several further samples had to be excluded from subsequent analysis due to inconclusive *HBA* (n = 41) or *HBB* (n = 1) genotyping results and missing values for anthropometry (n = 6), haemocytometry (n = 50), iron or inflammation biomarkers (n = 68). Characteristics of the included study population (n = 688) according to age ranges are given in Table 1.

**Table 1:** Demographic and anthropometric characteristics grouped by age

Characteristic	Overall n = 688	2-4y, n = 197	5-9y, n = 163	10-14y, n = 156	15 + y, n = 172
Sex					
Female	361 (52)	97 (49)	76 (47)	77 (49)	111 (65)
Body mass index^a^	16.0 (14.6, 18.5)	15.5 (14.6, 16.7)	14.6 (13.9, 15.4)	16.0 (15.0, 17.2)	19.6 (18.3, 21.9)
MUAC-for-age z score^a^	− 0.81 (− 1.46, − 0.14)	− 0.23 (− 0.78, 0.24)	− 0.91 (− 1.38, − 0.40)	− 1.46 (− 2.23, − 0.71)	− 1.30 (− 1.95, − 0.34)
	137	0	0	0	137
Haemoglobin (g/dl)^a^	11.40 (10.60, 12.20)	10.60 (9.50, 11.20)	11.10 (10.35, 11.80)	11.75 (11.10, 12.43)	12.40 (11.70, 13.50)
Haemoglobin group					
Nonanaemic	308 (45)	69 (35)	56 (34)	79 (51)	104 (60)
Anaemic	380 (55)	128 (65)	107 (66)	77 (49)	68 (40)
Haemoglobin genotype					
aa/aa with AA	356 (52)	104 (53)	83 (51)	82 (53)	87 (51)
-a/aa with AA	147 (21)	46 (23)	38 (23)	27 (17)	36 (21)
aa/aa with AC	98 (14)	27 (14)	22 (13)	23 (15)	26 (15)
-a/aa with AC	28 (4.1)	8 (4.1)	7 (4.3)	7 (4.5)	6 (3.5)
aa/aa with AS	24 (3.5)	2 (1.0)	8 (4.9)	8 (5.1)	6 (3.5)
-a/aa with AS	14 (2.0)	5 (2.5)	2 (1.2)	3 (1.9)	4 (2.3)
Rarer traits	21 (3.1)	5 (2.5)	3 (1.8)	6 (3.8)	7 (4.1)
Malnutrition					
Normal	419 (61)	162 (82)	92 (56)	49 (31)	116 (67)
Mild	185 (27)	33 (17)	55 (34)	59 (38)	38 (22)
Moderate	53 (7.7)	2 (1.0)	14 (8.6)	27 (17)	10 (5.8)
Severe	31 (4.5)	0 (0)	2 (1.2)	21 (13)	8 (4.7)
Parasitaemia					
Negative	304 (44)	120 (61)	51 (31)	30 (19)	103 (60)
Positive	384 (56)	77 (39)	112 (69)	126 (81)	69 (40)

Data are presented as n (%)

y: years; MUAC: mid upper-arm circumference; aa/aa with AA: wild type in alpha and beta genes; -a/aa with AA: 3.7 kb or 4.2 kb deletion; aa/aa with AC: haemoglobin C (heterozygous), -a/aa with AC = 3.7 kb deletion with haemoglobin C (heterozygous); aa/aa with AS: haemoglobin S (heterozygous); -a/aa with AS: 3.7 kb deletion with haemoglobin S (heterozygous), rarer traits (details in Additional file 1: Table S1, S2)

^a^Median (interquartile range)

### Haemoglobin genotypes

Haemoglobin genotyping was performed to identify thalassaemic and nonthalassaemic variants at the molecular level. This revealed a heterogeneous distribution, as shown in Table 1 and Additional file 1: Tables S1–S3), with the highest frequency being a single deletion in *HBA* combined with wild type *HBB* (-a/aa with AA).

Regarding exclusively the alpha genes, 70.6% (n = 417) wild type, 27.7% (n = 223) -a/aa and 1% (n = 8) anti-3.7 kb triplications were observed. Furthermore, two individuals with a 4.2 kb deletion were integrated into the respective 3.7 kb deletion group for further analysis, as they are considered phenotypically equivalent. The remaining 0.4% (n = 4) of the alpha mutations (FIL double deletion, THAI double deletion, haemoglobin H triple deletion, and 3.7 kb alpha deletion combined with triplication) were found only in single individuals each. One subject with the combination of 3.7 deletion and anti-3.7 triplication was included in the wild-type group for statistical analysis since this combination appears as a normal alpha phenotype.

Regarding exclusively the beta gene, 74.3% were wild type, 18.1% (n = 146) AC, 5.6% (n = 45) AS, 1.2% (n = 10) homozygous haemoglobin C (CC) and 0.7% (n = 6) compound heterozygous haemoglobin SC (SC).

Moreover, coinheritance of mutations in both genes occurs frequently for -a/aa with either AC (-a/aa with AC, 4.0%, n = 32) or AS (-a/aa with AS, 2.1%, n = 17). More complex mutations were observed at low frequencies (n < 10) and therefore grouped together into a rarer traits category. Further details and frequencies for each screened mutation are given in Additional file 1: Tables S1–S3.

**Fig. 1:**
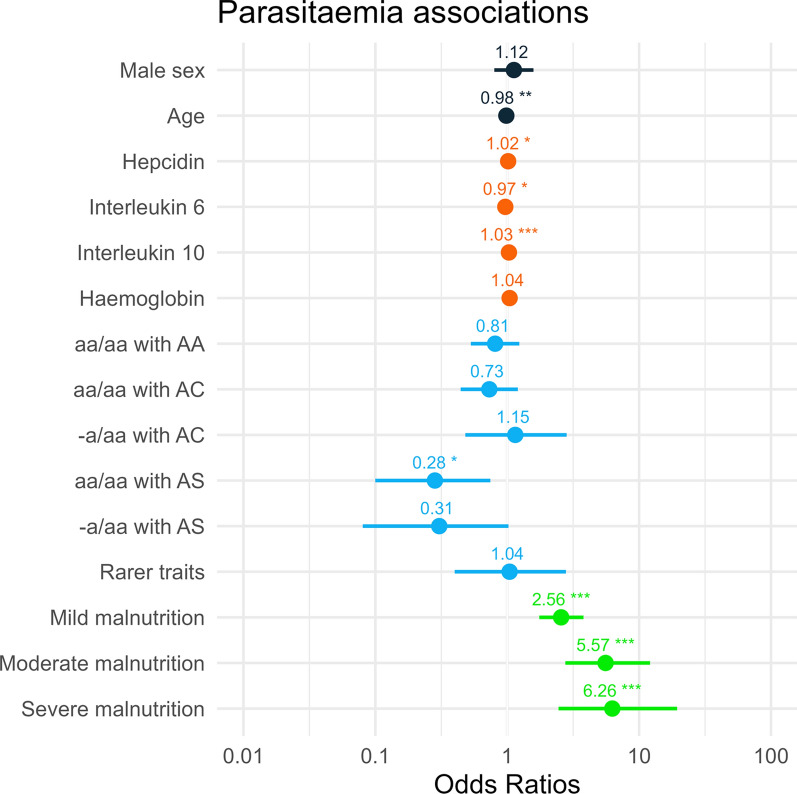
Forest plot of multivariable logistic regression results. Presence of parasitaemia was applied as the dichotomous response variable (more than or equal to 20 parasites per microlitre of whole blood were considered parasite positive). Included possible predictors: continuous parameters (age, hepcidin, cytokines, haemoglobin) and categorical variables (sex, haemoglobin genotype groups, malnutrition sub-categories). Colors group the covariates by subcategory (black: demographics, orange: continuous biomarkers, blue: haemoglobin genotypes, green: malnutrition). Significance codes: ***0.001; **0.01; *0.05; -a/aa with AA = single deletion of 3.7 kb or 4.2 kb, aa/aa with AC = haemoglobin C (heterozygous), -a/aa with AC = single deletion of 3.7 kb with haemoglobin C (heterozygous), aa/aa with AS = haemoglobin S (heterozygous), -a/aa with AS = single deletion of 3.7 kb with haemoglobin S (heterozygous); rarer traits = other genotype (details in Additional file 1: Tables S2, S3)

Univariate and multivariable logistic regression were performed in a subset of the dataset (n = 688) where values for all the covariates were present to assess the effect of various possible predictors (Fig. 1).

### Significant associations of AS groups with parasitaemia

**Fig. 2:**
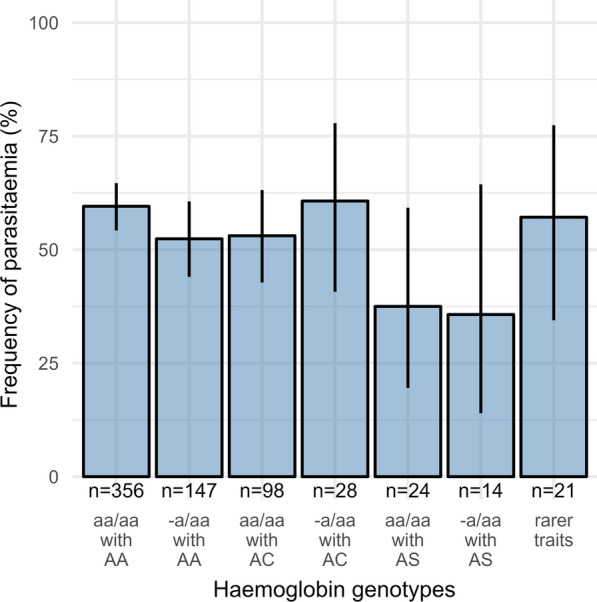
Proportions of positive parasitaemia in different haemoglobin genotypes (n = 688). Bars show percentages of positives in the respective genotype group, and lines represent individual 95% confidence interval. The basically reversed proportions in the aa/aa with AS and -a/aa with AS groups illustrate the effect of the sickle cell trait that is also present in asymptomatic parasite carriers. aa/aa with AA = wild type, -a/aa with AA = single deletion of 3.7 kb or 4.2 kb, aa/aa with AC = haemoglobin C (heterozygous), -a/aa with AC = single deletion of 3.7 kb with haemoglobin C (heterozygous), aa/aa with AS = haemoglobin S (heterozygous), -a/aa with AS = single deletion of 3.7 kb with haemoglobin S (heterozygous); rarer traits = other genotype (details in Additional file 1: Tables S2, S3)

aa/aa with AS was significantly associated with parasitaemia in the multivariable model (OR = 0.28, CI_95_: 0.10–0.74, p = 0.013). A marginally nonsignificant (OR = 0.31, CI_95_: 0.08–1.02, p = 0.063) effect was observed for the -a/aa with AS group. Furthermore, for these two groups, lower proportions of parasite positives were counted (Fig. 2). All other haemoglobin genotypes did not significantly differ from wild type in the probability of parasitaemia.

### Parasitaemia decreases with age

**Fig. 3:**
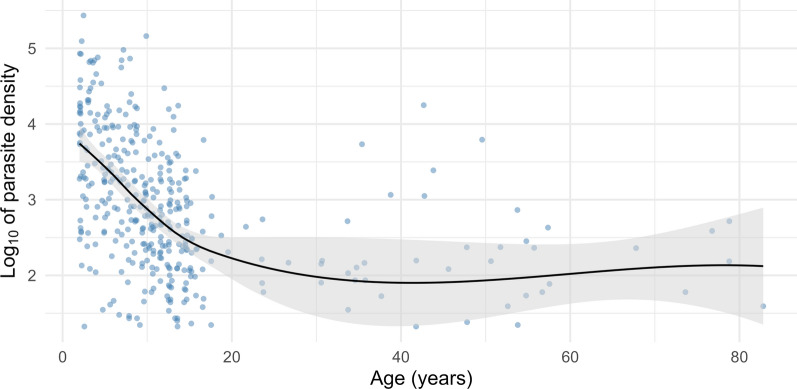
Logarithmic (log10) parasite density plotted versus age (n = 384). Aparasitaemic individuals (< 20 parasite/µL, n = 304) were excluded from this illustration. A prominent decrease in parasite density below 20 years in a linear fashion can be observed. The locally estimated scatterplot smoothed line is virtually constant for ages > 20

In the univariate analysis, younger age was a significant predictor of parasitaemia (OR = 0.98, CI_95_: 0.97–0.99, p < 0.001) and parasite density. Figure 3 illustrates the relationship between age and parasite density, which decreases with age until 20 years. Male sex was significantly predictive for parasitaemia in the univariate analysis (OR = 1.38, CI_95_: 1.02–1.86, p = 0.038) but was not when adjusted for possible confounders in the multivariable model (OR = 1.12, CI_95_: 0.80–1.58, p = 0.5).

### Parasitaemia and haematologic parameters

In the univariate analysis, haemoglobin concentration (OR = 0.90, CI_95_: 0.82–0.99, p = 0.025) and anaemia categories (OR = 0.74, CI_95_: 0.16–0.99, p = 0.047) were significantly associated with parasitaemia (Additional file 1: Figs. S1, S2). However, this did not hold true in the multivariable model. Two markers of microcytosis, mean corpuscular volume (OR = 0.99, CI_95_: 0.97–1.01, p = 0.3) and microcyte percentage (OR = 1.00, CI_95_: 0.99–1.02, p = 0.6), were not associated with the outcome.

### Parasitaemia, iron status and inflammation

Biomarkers of iron status, ferritin and soluble transferrin receptor, were not associated with parasitaemia in the multivariable analysis (OR = 1.00, CI_95_: 1.00–1.00, p = 0.15 and OR = 1.00, CI_95_: 0.99–1.01, p = 0.9). However, the multivariable model-adjusted odds ratio for hepcidin (OR = 1.02, CI_95_: 1.00–1.03, p = 0.043) was marginally significantly elevated. Concerning inflammatory parameters, both IL-6 (OR = 0.97, CI_95_: 0.93–0.99, p = 0.019) and IL-10 (OR = 1.03, CI_95_: 1.02–1.04, p < 0.001) showed significant effects in the adjusted model, while when fitted on its own, IL-6 had a borderline nonsignificant effect (OR = 1.02, CI_95_: 1.00–1.05, p = 0.067). Gametocytaemia was not significantly associated with parasitaemia in the univariate analysis (OR = 0.88, CI_95_: 0.55–1.41, p = 0.6). Further details are shown in Additional file 1: Figs. S3, S4.

### Parasitaemia and malnutrition

Calculation of z scores for the MUAC-for-age in younger subjects and BMI classification in adults lead to a surrogate categorization of energy sufficiency into normal, mild, moderate, and severe. Overall, malnutrition was prevalent at 39%, and particularly in individuals being five to nine and ten to 14 years old (Table 1).

**Fig. 4:**
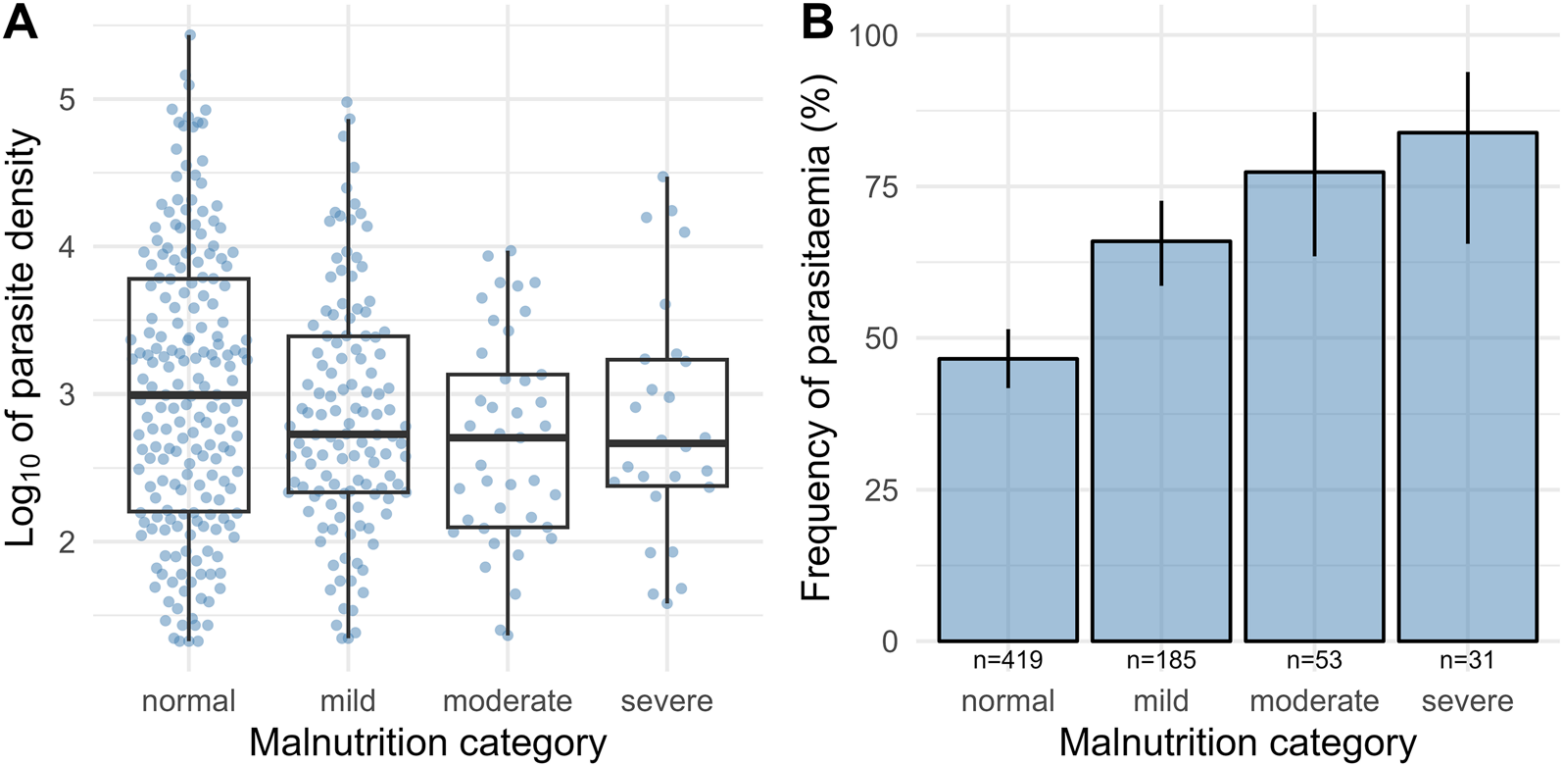
Relationship between asymptomatic malaria and nutrition, **A** boxplots of the four malnutrition categories (normal, n = 195; mild, n = 122; moderate, n = 41; severe, n = 26) showing lower parasitaemia in the three malnourished categories compared to the normal group; aparasitaemic individuals (< 20 parasites per microlitre, n = 304) were excluded from this illustration, **B** bar chart of parasite positives’ proportions with 95% confidence intervals in the malnutrition categories

In doing so, all three deficient categories, mild (OR = 2.56, CI_95_: 1.75–3.79, p < 0.001), moderate (OR = 5.57, CI_95_: 2.76–12.1, p < 0.001), and severe (OR = 6.26, CI_95_: 2.45–19.4, p < 0.001), were strong predictors of parasitaemia. The proportions of parasitaemia-positive individuals increased per category of malnutrition severity (Fig. 4B) suggesting a linear trend between parasitaemia and malnutrition categories (chi-squared test for trend, p < 0.001).

The results from comprehensive univariate and multivariable analyses are presented in Additional file 1: Tables S4–S6.

## Discussion

According to these findings, malarial parasitaemia was present in over half (n = 384) of otherwise healthy individuals (n = 688) from an endemic rural region in Burkina Faso during the high transmission (wet) season. Asymptomatic parasitaemia was attenuated in carriers of AS (aa/aa with AS and -a/aa with AS) mutations only but not in other haemoglobin genotypes (-a/aa with AA, aa/aa with AC, -a/aa with AC). Furthermore, it is showen that asymptomatic parasite carriage was strongly associated with malnutrition, and the effect size increased linearly with the severity of energy deficiency. To a minor extent, parasitaemia was also associated with other parameters, such as age and inflammation.

These findings complement the current knowledge on asymptomatic malaria parasitaemia in general and in haemoglobin variant carriers specifically.

### Asymptomatic parasitaemia and haemoglobin genotype

Moreover, the screened haemoglobin genotypes displayed a similar distribution as has been reported previously from Burkina Faso [28–30], although a considerably higher prevalence of alpha-thalassaemia trait carriers (-a/aa) was observed, which is likely explained by analytical-technical limitations and the different focus of previous studies [31]. However, no homozygotes for sickle cell disease (SS) were detected in this asymptomatic study population, despite previous reports from Burkina Faso showed an incidence of 1:167 [30, 32]. This could be explained by the fact that—without routine diagnosis and therapy—most SS children become symptomatic during the first year of life, and thus, drop out of the asymptomatic population, or die during the first year of life. Furthermore, no beta-thalassaemia variants were detected which could result from natural selection due to unfavourable pathologies if coinherited with AC or AS.

A considerable effect on malaria parasitaemia by common haemoglobin variants in asymptomatic subjects was expected, such as higher frequencies for individuals with aa/aa with AC and -a/aa with AC [9] or lower frequencies with aa/aa with AS and -a/aa with AS [11]. However, negative associations were only significant for aa/aa with AS and borderline nonsignificant for -a/aa with AS, whereas none were observed for other haemoglobin variants. While approximately 60% of wild-type individuals carry parasites, aa/aa with AS and -a/aa with AS genotype carriers show lower infection rates of approximately 40%. This is in line with their reported immediate protective effects [9–11, 33], although there is still no clear consensus on the underlying mechanisms [34–36]. Moreover, carriers of -a/aa have a reduced relative risk for severe malaria disease [10, 37], and negative epistasis with AS is only expected with deletions of more than one *HBA* gene [38]. These observations indicate that there is no negative epistasis detrimental to the protective effect of AS when coinherited with a single alpha deletion. These findings suggest that haemoglobin variants have a very limited additional effect on asymptomatic parasitaemia and immunological control of the parasite.

### Nongenetic parameters

Therefore, age, sex, iron parameters, inflammation and nutritional status were evaluated further in this large well-defined cohort to identify predictors of malaria parasitaemia in asymptomatic individuals.

Interestingly, despite parasitaemia peaking over 80% in the age group between 10 and 14 years, the parameter parasite density decreased with age linearly from one until 20 years old individuals. While the literature agrees that most clinical malaria cases occur during early life and in pregnant women [39], the linear quantitative relationship shown here has not yet been described in an exclusively asymptomatic cohort. In a longitudinal study [40], a similar effect was observed for the percentage of malaria attacks between 6 and 25 years of age. The decrease in parasite density may be explained by maturing immune and reticuloendothelial systems and thereby limited parasite proliferation [41].

As previously outlined, haemoglobin concentration as a robust marker for general anaemia classification was significantly associated with parasitaemia only in the univariate analysis and not in the multivariable model. Since all-cause anaemia is very common in individuals of the investigated area, haemoglobin alone might not serve as surrogate predictor of parasitaemia.

Associations of IL-6 and IL-10 with parasitaemia may indicate an ongoing inflammatory response in infected individuals. Both were significantly higher in parasitaemic compared to aparasitaemic individuals. IL-6 is known to play a pro-inflammatory role, and IL-10 plays an anti-inflammatory role during inflammation. In contrast, IL-10 was proposed to influence the susceptibility to coinfections in asymptomatic malaria parasitaemia [42], which might result from a brittle equilibrium of inflammatory agents slightly in favour of resolution.

In contrast, the iron biomarkers ferritin (storage iron) and soluble transferrin receptor (functional iron supply) were not associated with parasitaemia in the current study. This relationship of parasitaemia and iron homeostasis is discussed controversially in the literature [43–45]. The reported interaction of ferritin and inflammation in asymptomatic malaria parasitaemia might partially be explained by the nature of ferritin as an acute phase protein but are not consistent with this study’s findings. On the other hand, chronic low-grade inflammation may affect health in the long term and lead to decreased iron supply and subsequently haemoglobin levels, which is a public health concern in general [46].

A central regulator of iron homeostasis, hepcidin, was marginally associated with parasitaemia (OR = 1.02, CI_95_: 1.00–1.03, p = 0.043). Control of peripheral hepcidin expression in macrophages is influenced by IL-10 according to in vitro studies [47], but the main source expressing the bactericidal peptide, hepatocytes, remains unaffected. This could explain the minor effect that has been observed in asymptomatic individuals with low-grade inflammation according to laboratory data and agrees with a previous report [48]. While weakly associated, hepcidin still inhibits iron supply into circulation by sequestration into stores, which is important for susceptibility to coinfections [49] and disease progression, as described elsewhere [50–52].

However, iron availability is further determined by nutritional intake. Malnutrition was an important predictor of parasitaemia in the presented findings and showed a large effect that increased with severity. This differs from a study, not observing an association between undernutrition and asymptomatic parasitaemia in Ghana [53]. A recent meta-analysis [13] investigating the inverse relationship of malaria and micronutrients concluded on the necessity of normalization of nutritional assessments to individuals with malaria parasitaemia, especially in asymptomatic subjects.

Furthermore, inflammation, iron homeostasis, and nutritional impairment are dependent on each other, and mere supplementation with iron will not resolve infection, but might even worsen the outcome [54]. On the other hand, it is well-known that malnutrition compromises inflammatory responses and increases susceptibility to different infections. Another recent meta-analysis confirmed that chronic malnutrition is consistently associated with the severity of malaria [16]. The linear increase in parasitaemia with malnutrition severity emphasises that even small advances ameliorating energy deficiency should benefit efforts against malaria transmission probability.

These data indicate that nutritional interventions are needed to further study the effect on asymptomatic parasitaemia and as such represent a possible tool to reduce the burden of malaria.

### Strengths and limitations

An essential strength of these investigation is that genotyping was performed on a molecular level, while previous studies from Burkina Faso focused on the determination of specific *HBB* variants at the protein level [28, 55] and indirect estimates of alpha-thalassaemia mutations. Therefore, zygosity and deletional or nondeletional character of the defect were previously not distinguished [28, 55]. A large age range was covered by this study. Parasite density and parasitaemia were determined by state-of-the-art technology.

The sample size did not justify analysis of the observed proportions of the rarer traits and did not allow to analyse potential effects of these rare genotypes. The analysis of symptomatic or severe malaria disease was not subject to this study, but the focus was on asymptomatic individuals. Moreover, subclinical or symptomatic infestation with infectious agents other than malaria parasites were not assessed and their influence on cytokine levels could not be tested.

## Conclusion

Asymptomatic malaria parasitaemia is observed frequently in the rural population of Burkina Faso during the high transmission season. Additionally, haemoglobin variants are very common in this region, but apart from genotypes AS and -a/aa with AS, no relationship with asymptomatic parasitaemia was observed. These data suggest a significant relationship between asymptomatic parasitaemia with increasing malnutrition. Interventions to improve the overall nutritional status and energy sufficiency in children and adolescents in malaria-endemic areas are warranted and should include exploring its effect on *P. falciparum* parasitaemia.

### Supplementary Information

**Additional file 1: Table S1.** Frequency of combined haemoglobin genotypes *HBA1/HBA2 *and *HBB* genes (total n = 805). **Table S2.** Frequency of all 21 haemoglobin genotypes covered by the strip assays (Viennalab alpha-Globin for HBA, total samples n = 805), two individuals showed co-inheritance of more than one mutation. **Table S3.** Frequency of all 22 haemoglobin genotypes covered by the strip assay (Viennalab beta-Globin MED for HBB, total samples n = 805), 16 individuals showed co-inheritance of more than one mutation. **Table S4.** Univariate logistic regression results of parasitaemia as response variable. **Table S5.** Univariate linear regression results of parasite density as response variable. **Table S6.** Multivariable logistic regression of parasitaemia as response variable (total n = 688). The overall probability of parasitaemia was 56%. **Figure S1.** Relationship of anaemia and asymptomatic malaria parasitaemia (log10 of parasite density). (A) A lower parasite density in the non-anaemic group is documented (p < 0.001, n = 384). (B) Lower haemoglobin concentrations are documented for the aparasitaemic group (p = 0.02, n = 688). **Figure S2.** Parasitaemia positive frequency for the basic anaemia categories (age-related total haemoglobin cut-offs) is plotted in bars and lines describing 95% CI from binomial distribution. A similar difference as in Fig. S3 (A) is observed as the parasitaemia and parasite density both appear lower in the non-anaemic group. **Figure S3.** Inflammation marker concentrations (log10 transformed) are illustrated and grouped by qualitative parasitaemia categories (n = 688). Highly significant differences are observed for all the parameters. **Figure S4.** Positive parasitaemia frequency for the gametocytaemia categories (cut-off of 1.3/µL was previously defined in the literature, [27]) is plotted in bars and lines with 95% CI from binomial distribution as lines. Differences are non-significant. Gametocytaemia is similarly prevalent in the non-parasitised group in this investigated endemic region. **Figure S5.** Body mass index is illustrated in a dot plot with box plots overlayed grouped by categories of nutritional status (n = 688). Individuals were categorised depending on their age, and upper-arm circumference or body mass index. For the group < 19 years malnutrition status was determined by MUAC-for-age z-score and for the adult group of 20 years and older body mass index classification was used. Both variables were calculated and previously published cut-offs applied.

**Additional file 2.** A CSV with the analysed dataset that can be used to reproduce the results of this study.

## Data Availability

The datasets used and/or analysed during the current study are available in the supplement or from the corresponding author on reasonable request.

## Abbreviations

-a/aa: 3.7 kb deletion in the alpha gene
-a/aa with AC: Coinheritance single 3.7 kb deletion on the alpha gene and haemoglobin C on the beta gene
-a/aa with AS: Coinheritance single 3.7 kb deletion on the alpha gene and haemoglobin S on the beta gene
aa/aa with AA: Wild type haemoglobin alpha and beta genes
aa/aa with AC: Coinheritance wild type on the alpha gene and haemoglobin C on the beta gene
aa/aa with AS: Coinheritance wild type on the alpha gene and haemoglobin S on the beta gene
AC: Heterozygous haemoglobin C mutation on the beta gene
AS: Heterozygous haemoglobin S mutation on the beta gene
BMI: Body mass index
CC: Homozygous haemoglobin C mutation on the beta gene
ELISA: Enzyme-linked immunosorbent assay
*HBA*: Haemoglobin alpha chain gene
*HBB*: Haemoglobin beta chain gene
IL-10: Interleukin 10
IL-6: Interleukin 6
MUAC: Mid upper-arm circumference
NOVAC: Nonspecific Effects of Vaccination on Mortality and Morbidity
OR: Odds ratio
SC: Compound heterozygous haemoglobin S and C mutation on the beta gene
SS: Homozygous haemoglobin S mutation on the beta gene
