# Supplementary material for: Exploring the host factors affecting asymptomatic Plasmodium falciparum infection: insights from a rural Burkina Faso study
Source: Malar J. 2023 Sep 1;22:252. doi: 10.1186/s12936-023-04686-0 (PMC10474782; doi:10.1186/s12936-023-04686-0)
Supplement: Supplementary file 1 — Additional file 1: Table S1. Frequency of combined haemoglobin genotypes HBA1/HBA2 and HBB genes (total n = 805). Table S2. Frequency of all 21 haemoglobin genotypes covered by the strip assays (Viennalab alpha-Globin for HBA, total samples n = 805), two individuals showed co-inheritance of more than one mutation. Table S3. Frequency of all 22 haemoglobin genotypes covered by the strip assay (Viennalab beta-Globin MED for HBB, total samples n = 805), 16 individuals showed co-inheritance of more than one mutation. Table S4. Univariate logistic regression results of parasitaemia as response variable. Table S5. Univariate linear regression results of parasite density as response variable. Table S6. Multivariable logistic regression of parasitaemia as response variable (total n = 688). The overall probability of parasitaemia was 56%. Figure S1. Relationship of anaemia and asymptomatic malaria parasitaemia (log10 of parasite density). (A) A lower parasite density in the non-anaemic group is documented (p < 0.001, n = 384). (B) Lower haemoglobin concentrations are documented for the aparasitaemic group (p = 0.02, n = 688). Figure S2. Parasitaemia positive frequency for the basic anaemia categories (age-related total haemoglobin cut-offs) is plotted in bars and lines describing 95% CI from binomial distribution. A similar difference as in Fig. S3 (A) is observed as the parasitaemia and parasite density both appear lower in the non-anaemic group. Figure S3. Inflammation marker concentrations (log10 transformed) are illustrated and grouped by qualitative parasitaemia categories (n = 688). Highly significant differences are observed for all the parameters. Figure S4. Positive parasitaemia frequency for the gametocytaemia categories (cut-off of 1.3/µL was previously defined in the literature, [27]) is plotted in bars and lines with 95% CI from binomial distribution as lines. Differences are non-significant. Gametocytaemia is similarly prevalent in the non-parasitised group in [file 12936_2023_4686_MOESM1_ESM.docx]

Supplementary tables

Table S1: Frequency of combined haemoglobin genotypes *HBA1/HBA2* and *HBB* genes (total n=805).

| Haemoglobin genotype | Frequency (n) | Rel. frequency (%) |
| --- | --- | --- |
| Wild type | 416 | 51.7 |
| Single deletion of 3.7 kb | 171 | 21.2 |
| Haemoglobin C (heterozygous) | 112 | 13.9 |
| Single deletion 3.7 kb with haemoglobin C (heterozygous) | 32 | 4 |
| Haemoglobin S (heterozygous) | 28 | 3.5 |
| Single deletion 3.7 kb with haemoglobin S (heterozygous) | 17 | 2.1 |
| Haemoglobin C (homozygous) | 8 | 1 |
| Triplication or quadruplication anti-3.7 | 7 | 0.9 |
| Haemoglobin S and C (compound heterozygous) | 4 | 0.5 |
| Single deletion of 3.7 kb with haemoglobin S and C (compound heterozygous) | 2 | 0.2 |
| Double deletion (FIL) | 1 | 0.1 |
| Triple deletion (FIL) and 3.7 kb | 1 | 0.1 |
| Double deletion (THAI) | 1 | 0.1 |
| Single deletion 3.7 kb and triplication anti-3.7 | 1 | 0.1 |
| Single deletion of 3.7 kb with haemoglobin C (homozygous) | 1 | 0.1 |
| Single deletion of 4.2 kb with haemoglobin C (homozygous) | 1 | 0.1 |
| Single deletion of 4.2 kb with haemoglobin C (heterozygous) | 1 | 0.1 |
| Triplication or quadruplication anti-3.7 with haemoglobin C (heterozygous) | 1 | 0.1 |

Table S2: Frequency of all 21 haemoglobin genotypes covered by the strip assays (Viennalab alpha-Globin for HBA, total samples n=805), two individuals showed co-inheritance of more than one mutation.

| *HBA* genotype | Frequency (n) | Rel. frequency (%) |
| --- | --- | --- |
| Wild type | 568 | 70.6 |
| Single deletion of 3.7 kb | 225 | 27.9 |
| Triplication or quadruplication anti-3.7 | 9 | 1.1 |
| Single deletion of 4.2 kb | 2 | 0.2 |
| Double deletion (THAI) | 1 | 0.1 |
| Double deletion (FIL) | 2 | 0.2 |
| Double deletion (MED) | n/a | n/a |
| Double deletion (SEA) | n/a | n/a |
| Double deletion of 20.5 kb | n/a | n/a |
| α1 cd 14 [TGG>TAG] | n/a | n/a |
| α1 cd 59 [GGC>GAC] (Hb Adana) | n/a | n/a |
| α2 init cd [ATG>ACG] | n/a | n/a |
| α2 cd 19 [-G] | n/a | n/a |
| α2 IVS1 [-5nt] | n/a | n/a |
| α2 cd 59 [GGC>GAC] | n/a | n/a |
| α2 cd 125 [CTG>CCG] (Hb Quong Sze) | n/a | n/a |
| α2 cd 142 [TAA>CAA] (Hb Constant Spring) | n/a | n/a |
| α2 cd 142 [TAA>AAA] (Hb Icaria) | n/a | n/a |
| α2 cd 142 [TAA>TAT] (Hb Pakse) | n/a | n/a |
| α2 cd 142 [TAA>TCA] (Hb Koya Dora) | n/a | n/a |
| α2 poly A-1 [AATAAA-AATAAG] | n/a | n/a |
| α2 poly A-2 [AATAAA-AATGAA] | n/a | n/a |

*HBA*, haemoglobin alpha chain gene; n/a, not applicable

Table S3: Frequency of all 22 haemoglobin genotypes covered by the strip assay (Viennalab beta-Globin MED for HBB, total samples n=805), 16 individuals showed co-inheritance of more than one mutation.

| *HBB* genotype | Frequency (n) | Rel. frequency (%) |
| --- | --- | --- |
| Wild type | 598 | 74.3 |
| codon 6 [G>A] Haemoglobin C | 162 | 20 |
| codon 6 [A>T] Haemoglobin S | 51 | 6.3 |
| - 101 [C>T] | n/a | n/a |
| - 87 [C>G] | n/a | n/a |
| - 30 [T>A] | n/a | n/a |
| codon 5 [-CT] | n/a | n/a |
| codon 6 [-A] | n/a | n/a |
| codon 8 [-AA] | n/a | n/a |
| codon 8/9 [+G] | n/a | n/a |
| codon 15 [TGG>TGA] | n/a | n/a |
| codon 27 [G>T] Knossos | n/a | n/a |
| IVS 1.1 [G>A] | n/a | n/a |
| IVS 1.5 [G>C] | n/a | n/a |
| IVS 1.6 [T>C] | n/a | n/a |
| IVS 1.110 [G>A] | n/a | n/a |
| IVS 1.116 [T>G] | n/a | n/a |
| IVS 1.130 [G>C] | n/a | n/a |
| codon 39 [C>T] | n/a | n/a |
| codon 44 [-C] | n/a | n/a |
| IVS 2.1 [G>A] | n/a | n/a |
| IVS 2.745 [C>G] | n/a | n/a |
| IVS 2.848 [C>A] | n/a | n/a |

*HBB*, haemoglobin beta chain gene; n/a, not applicable

Table S4: Univariate logistic regression results of parasitaemia as response variable.

| Characteristic | N | OR | 95% CI | p-value |
| --- | --- | --- | --- | --- |
| Sex | 688 |  |  |  |
| Female |  | — | — |  |
| Male |  | 1.38 | 1.02, 1.86 | 0.038 |
| Age | 688 | 0.98 | 0.97, 0.99 | <0.001 |
| Soluble transferrin receptor concentration | 688 | 1.00 | 0.99, 1.01 | 0.9 |
| Hepcidin concentration | 688 | 1.03 | 1.01, 1.04 | <0.001 |
| Ferritin concentration | 688 | 1.00 | 1.00, 1.00 | 0.15 |
| TNF-alpha concentration | 688 | 1.02 | 1.01, 1.04 | <0.001 |
| Interleukin 6 concentration | 688 | 1.02 | 1.00, 1.05 | 0.067 |
| Interleukin 10 concentration | 688 | 1.03 | 1.02, 1.04 | <0.001 |
| Interferon gamma concentration | 688 | 1.00 | 0.98, 1.02 | 0.7 |
| Gametocyte concentration | 688 | 1.03 | 1.02, 1.06 | 0.003 |
| Rel. gametocyte concentration | 688 | 1.15 | 1.07, 1.29 | 0.003 |
| Red blood cell count | 688 | 0.79 | 0.61, 1.01 | 0.066 |
| Haemoglobin concentration | 688 | 0.90 | 0.82, 0.99 | 0.025 |
| Hematocrit | 688 | 0.96 | 0.93, 0.99 | 0.011 |
| Mean corpuscular volume | 688 | 0.99 | 0.97, 1.01 | 0.3 |
| Mean corpuscular haemoglobin | 688 | 0.97 | 0.92, 1.03 | 0.3 |
| Mean corpuscular haemoglobin concentration | 688 | 1.01 | 0.92, 1.10 | >0.9 |
| Red cell distribution width coefficient of variation | 688 | 0.98 | 0.92, 1.04 | 0.4 |
| Percentage of microcytes | 688 | 1.00 | 0.99, 1.02 | 0.6 |
| Percentage of macrocytes | 688 | 1.00 | 0.87, 1.15 | >0.9 |
| *HBA/HBB* | 688 |  |  |  |
| aa/aa with AA |  | — | — |  |
| -a/aa with AA |  | 0.75 | 0.51, 1.10 | 0.14 |
| aa/aa with AC |  | 0.77 | 0.49, 1.21 | 0.2 |
| -a/aa with AC |  | 1.05 | 0.48, 2.37 | >0.9 |
| aa/aa with AS |  | 0.41 | 0.17, 0.94 | 0.039 |
| -a/aa with AS |  | 0.38 | 0.11, 1.12 | 0.086 |
| Rarer traits |  | 0.91 | 0.37, 2.27 | 0.8 |
| Gametocytaemia | 688 |  |  |  |
| Negative |  | — | — |  |
| Positive |  | 0.88 | 0.55, 1.41 | 0.6 |
| Malnutrition | 688 |  |  |  |
| Normal |  | — | — |  |
| Mild |  | 2.22 | 1.56, 3.20 | <0.001 |
| Moderate |  | 3.92 | 2.07, 8.00 | <0.001 |
| Severe |  | 5.97 | 2.44, 17.9 | <0.001 |
| Anaemia | 688 |  |  |  |
| Non-anaemic |  | — | — |  |
| Anaemic |  | 1.36 | 1.01, 1.84 | 0.047 |
| OR = Odds Ratio, CI = Confidence Interval, *HBA*, haemoglobin alpha chain gene; *HBB*, haemoglobin beta chain gene aa/aa with AA = wild type, -a/aa with AA = single deletion of 3.7 kb or 4.2 kb, aa/aa with AC = haemoglobin C (heterozygous), -a/aa with AC = single deletion of 3.7 kb with haemoglobin C (heterozygous), aa/aa with AS = haemoglobin S (heterozygous), -a/aa with AS = single deletion of 3.7 kb with haemoglobin S (heterozygous)  Detailed composition of rarer traits category in supplementary table S2 & S3 | | | | |

Table S5: Univariate linear regression results of parasite density as response variable.

| Characteristic | N | Beta | 95% CI | p-value |
| --- | --- | --- | --- | --- |
| Sex | 688 |  |  |  |
| Female |  | — | — |  |
| Male |  | 0.41 | 0.10, 0.71 | 0.010 |
| Age | 688 | -0.02 | -0.03, -0.02 | <0.001 |
| Soluble transferrin receptor concentration | 688 | 0.01 | 0.00, 0.02 | 0.2 |
| Hepcidin concentration | 688 | 0.03 | 0.02, 0.04 | <0.001 |
| Ferritin concentration | 688 | 0.00 | 0.00, 0.00 | 0.017 |
| TNF-alpha concentration | 688 | 0.02 | 0.01, 0.03 | <0.001 |
| Interleukin 6 concentration | 688 | 0.03 | 0.02, 0.05 | <0.001 |
| Interleukin 10 concentration | 688 | 0.01 | 0.01, 0.01 | <0.001 |
| Interferon gamma concentration | 688 | 0.01 | -0.01, 0.02 | 0.5 |
| Gametocyte concentration | 688 | 0.00 | 0.00, 0.01 | <0.001 |
| Rel. gametocyte concentration | 688 | 0.02 | 0.01, 0.03 | <0.001 |
| Red blood cell count | 688 | -0.46 | -0.71, -0.20 | <0.001 |
| Haemoglobin concentration | 688 | -0.22 | -0.31, -0.13 | <0.001 |
| Hematocrit | 688 | -0.08 | -0.11, -0.05 | <0.001 |
| Mean corpuscular volume | 688 | -0.02 | -0.04, 0.00 | 0.026 |
| Mean corpuscular haemoglobin | 688 | -0.06 | -0.12, -0.01 | 0.018 |
| Mean corpuscular haemoglobin concentration | 688 | -0.04 | -0.14, 0.06 | 0.4 |
| Red cell distribution width coefficient of variation | 688 | 0.04 | -0.02, 0.10 | 0.2 |
| Percentage of microcytes | 688 | 0.01 | 0.00, 0.03 | 0.048 |
| Percentage of macrocytes | 688 | -0.03 | -0.16, 0.11 | 0.7 |
| *HBA/HBB* | 688 |  |  |  |
| aa/aa with AA |  | — | — |  |
| -a/aa with AA |  | -0.23 | -0.62, 0.17 | 0.3 |
| aa/aa with AC |  | -0.22 | -0.68, 0.24 | 0.3 |
| -a/aa with AC |  | 0.10 | -0.69, 0.89 | 0.8 |
| aa/aa with AS |  | -0.94 | -1.8, -0.09 | 0.031 |
| -a/aa with AS |  | -1.1 | -2.2, -0.04 | 0.041 |
| Rarer traits |  | -0.02 | -0.92, 0.89 | >0.9 |
| Gametocytaemia | 688 |  |  |  |
| Negative |  | — | — |  |
| Positive |  | -0.17 | -0.65, 0.31 | 0.5 |
| Malnutrition | 688 |  |  |  |
| Normal |  | — | — |  |
| Mild |  | 0.67 | 0.32, 1.0 | <0.001 |
| Moderate |  | 0.97 | 0.39, 1.6 | 0.001 |
| Severe |  | 1.3 | 0.58, 2.1 | <0.001 |
| Anaemia | 688 |  |  |  |
| Non-anaemic |  | — | — |  |
| Anaemic |  | 0.51 | 0.20, 0.82 | 0.001 |
| OR = Odds Ratio, CI = Confidence Interval, *HBA*, haemoglobin alpha chain gene; *HBB*, haemoglobin beta chain gene aa/aa with AA = wild type, -a/aa with AA = single deletion of 3.7 kb or 4.2 kb, aa/aa with AC = haemoglobin C (heterozygous), -a/aa with AC = single deletion of 3.7 kb with haemoglobin C (heterozygous), aa/aa with AS = haemoglobin S (heterozygous), -a/aa with AS = single deletion of 3.7 kb with haemoglobin S (heterozygous)  Detailed composition of rarer traits category in supplementary table S2 & S3 | | | | |

Table S6: Multivariable logistic regression of parasitaemia as response variable (total n=688). The overall probability of parasitaemia was 56%.

| Characteristic | N | OR | 95% CI | p-value |
| --- | --- | --- | --- | --- |
| Sex |  |  |  |  |
| Female | 361 | — | — |  |
| Male | 327 | 1.12 | 0.80, 1.58 | 0.5 |
| Age |  | 0.98 | 0.97, 0.99 | 0.003 |
| Hepcidin concentration |  | 1.02 | 1.00, 1.03 | 0.043 |
| Interleukin 6 |  | 0.97 | 0.93, 0.99 | 0.019 |
| Interleukin 10 |  | 1.03 | 1.02, 1.04 | <0.001 |
| Haemoglobin concentration |  | 1.04 | 0.93, 1.17 | 0.5 |
| *HBA/HBB* |  |  |  |  |
| aa/aa with AA | 417 | — | — |  |
| -a/aa with AA | 171 | 0.81 | 0.53, 1.24 | 0.3 |
| aa/aa with AC | 112 | 0.73 | 0.44, 1.20 | 0.2 |
| -a/aa with AC | 33 | 1.15 | 0.48, 2.82 | 0.8 |
| aa/aa with AS | 28 | 0.28 | 0.10, 0.74 | 0.013 |
| -a/aa with AS | 17 | 0.31 | 0.08, 1.02 | 0.063 |
| Rarer traits | 27 | 1.04 | 0.40, 2.79 | >0.9 |
| Malnutrition |  |  |  |  |
| Normal | 419 | — | — |  |
| Mild | 185 | 2.56 | 1.75, 3.79 | <0.001 |
| Moderate | 53 | 5.57 | 2.76, 12.1 | <0.001 |
| Severe | 31 | 6.26 | 2.45, 19.4 | <0.001 |
| OR = Odds Ratio, CI = Confidence Interval, *HBA*, haemoglobin alpha chain gene; *HBB*, haemoglobin beta chain gene aa/aa with AA = wild type, -a/aa with AA = single deletion of 3.7 kb or 4.2 kb, aa/aa with AC = haemoglobin C (heterozygous), -a/aa with AC = single deletion of 3.7 kb with haemoglobin C (heterozygous), aa/aa with AS = haemoglobin S (heterozygous), -a/aa with AS = single deletion of 3.7 kb with haemoglobin S (heterozygous)  Detailed composition of rarer traits category in supplementary table S2 & S3 | | | | |

Supplementary figures


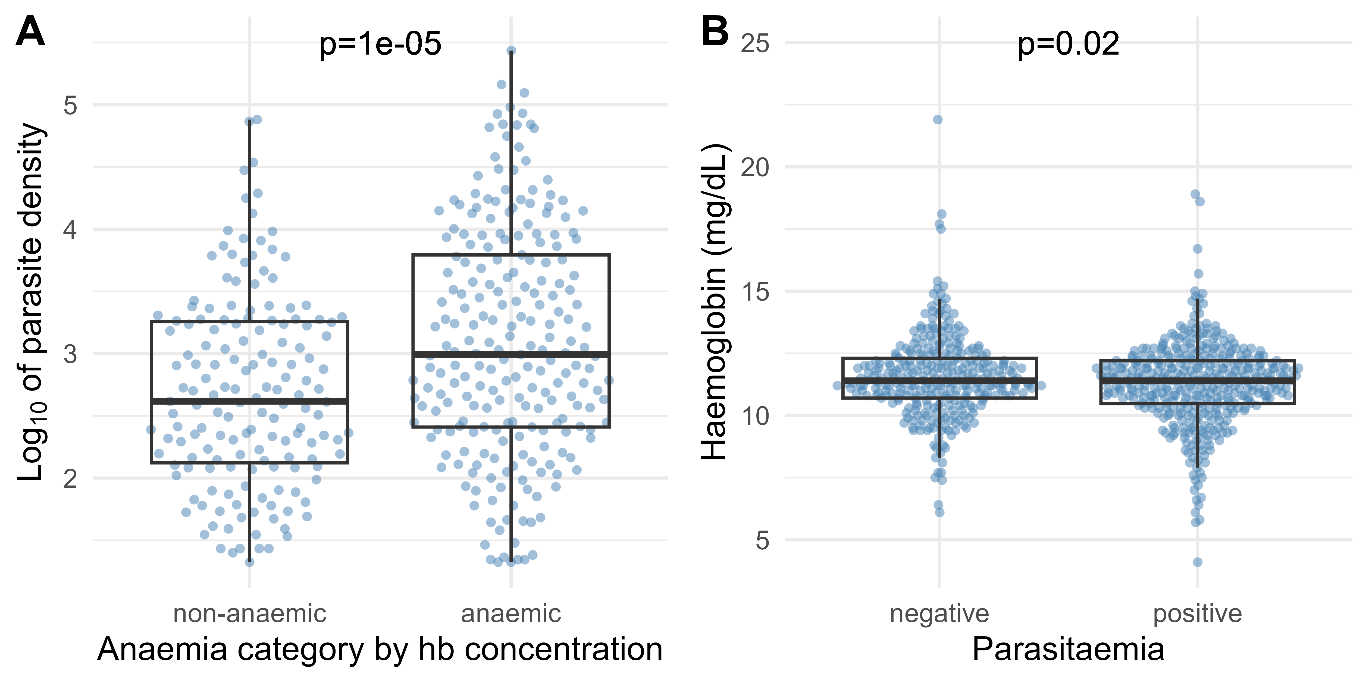


Figure S1. Relationship of anaemia and asymptomatic malaria parasitaemia (log10 of parasite density). (A) A lower parasite density in the non-anaemic group is documented (p<0.001, n=384). (B) Lower haemoglobin concentrations are documented for the aparasitaemic group (p=0.02, n=688).


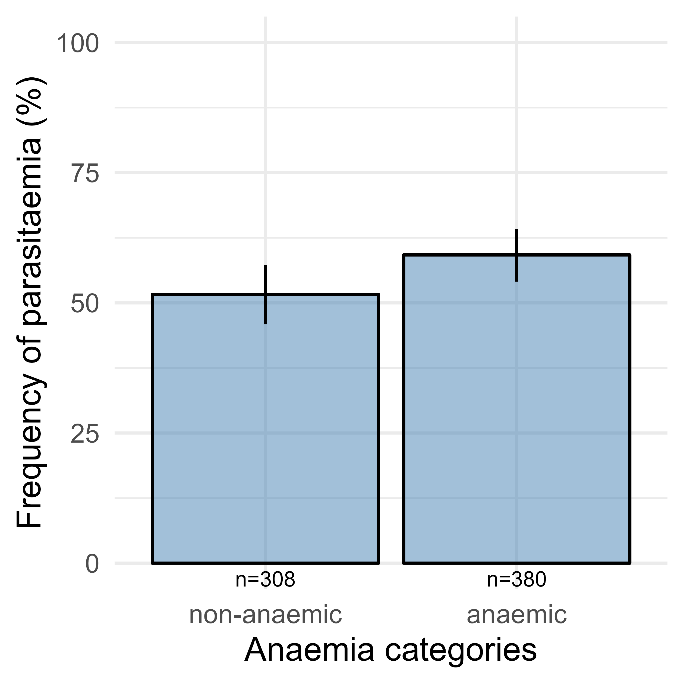


Figure S2: Parasitaemia positive frequency for the basic anaemia categories (age-related total haemoglobin cut-offs) is plotted in bars and lines describing 95% CI from binomial distribution. A similar difference as in Figure S3 (A) is observed as the parasitaemia and parasite density both appear lower in the non-anaemic group.


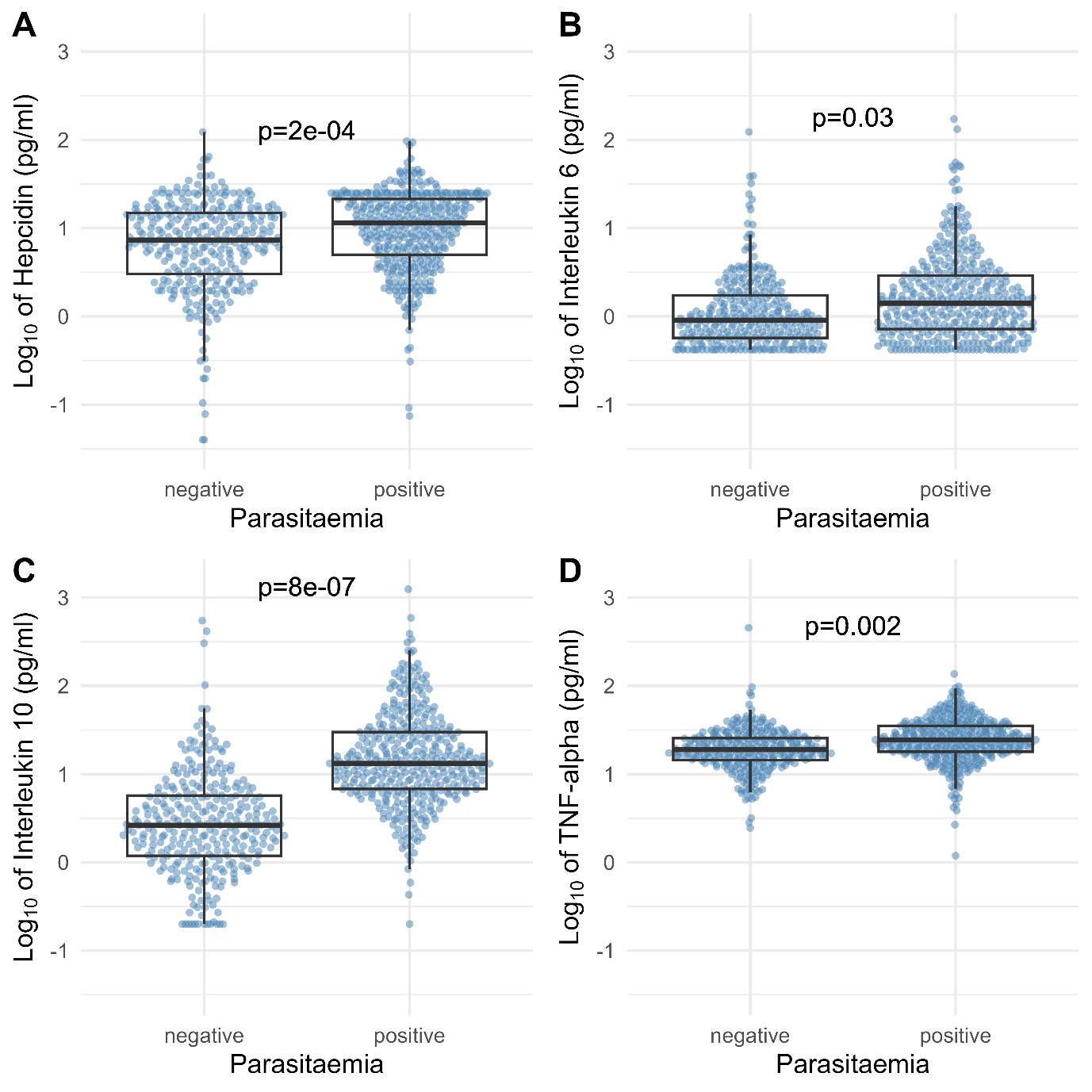


Figure S3: Inflammation marker concentrations (log10 transformed) are illustrated and grouped by qualitative parasitaemia categories (n=688). Highly significant differences are observed for all the parameters.


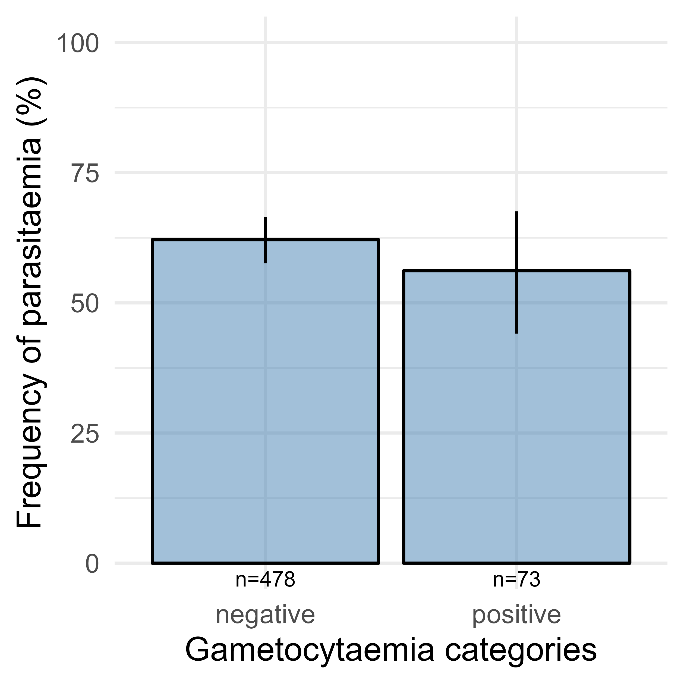


Figure S4: Positive parasitaemia frequency for the gametocytaemia categories (cut-off of 1.3 /µL was previously defined in the literature, Post *et al.* 2019) is plotted in bars and lines with 95% CI from binomial distribution as lines. Differences are non-significant. Gametocytaemia is similarly prevalent in the non-parasitised group in this investigated endemic region.


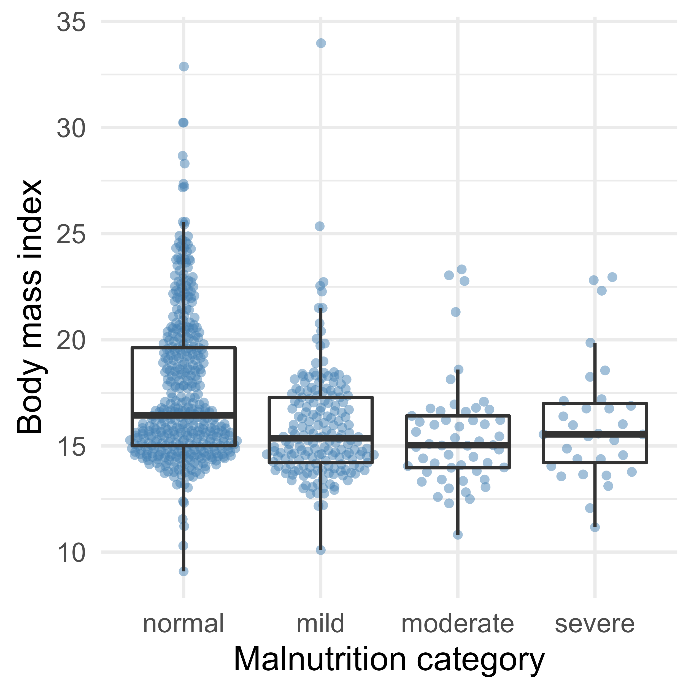


Figure S5. Body mass index is illustrated in a dot plot with box plots overlayed grouped by categories of nutritional status (n=688). Individuals were categorised depending on their age, and upper-arm circumference or body mass index. For the group <19 years malnutrition status was determined by MUAC-for-age z-score and for the adult group of 20 years and older body mass index classification was used. Both variables were calculated and previously published cut-offs applied.
